# Array-based genotyping and expression analysis of barley cv. Maythorpe and Golden Promise

**DOI:** 10.1186/1471-2164-8-87

**Published:** 2007-03-30

**Authors:** Harkamal Walia, Clyde Wilson, Pascal Condamine, Abdelbagi M Ismail, Jin Xu, Xinping Cui, Timothy J Close

**Affiliations:** 1 Department of Botany and Plant Sciences, University of California, Riverside, CA, USA; 2 USDA-ARS, U. S. Salinity Laboratory, Riverside, CA, USA; 3 Crop and Environmental Sciences Division, International Rice Research Institute, Philippines; 4 Department of Statistics, East China Normal University, Shanghai, China; 5 Department of Statistics, University of California, Riverside, CA, USA; 6 Genome Center, University of California, Davis, CA 95616, USA

## Abstract

**Background:**

Golden Promise is a salt-tolerant spring barley closely related to Maythorpe. Salt tolerance in Golden Promise has been attributed to a single mutation at the *Ari-e *locus (on 5H) resulting from irradiation of Maythorpe. Golden Promise accumulates lower shoot Na^+ ^compared to Maythorpe when growing under saline conditions. This study focused on elucidating the genetic basis and mechanisms involved in this difference.

**Results:**

The level of polymorphism between the two genotypes was explored using the Barley1 GeneChip for single feature polymorphisms (SFPs) and an oligonucleotide pool assay for single nucleotide polymorphisms (SNPs). Polymorphism analyses revealed three haplotype blocks spanning 6.4 cM on chromosome 1H, 23.7 cM on chromosome 4H and 3.0 cM on 5H. The Barley1 GeneChip was used to examine transcript abundance in different tissues and stages during development. Several genes within the polymorphic haplotype blocks were differentially regulated. Additionally, a more global difference in the jasmonic acid pathway regulation was detected between the two genotypes.

**Conclusion:**

The results confirm that Golden Promise and Maythorpe are genetically very closely related but establish that they are not isogenic, as previously reported, due to three polymorphic haplotype blocks. Transcriptome analysis indicates that the response of the two genotypes to salinity stress is quite different. Additionally, the response to salinity stress in the roots and shoot tissue is strikingly different.

## Background

Barley (*Hordeum vulgare *L.) is rated as a salt-tolerant member of the tribe *Triticeae *on the basis of grain yield in saline environments [1]. Salt tolerance in *Triticeae *is generally associated with Na^+ ^ion exclusion during growth under saline conditions [2,3]. Considerable genetic variation exists in salt tolerance with respect to Na^+ ^ion exclusion in barley as well as in *Triticeae *in general. Barley cultivar, Golden Promise was reported to be a gamma-ray induced mutant of cultivar Maythorpe [4]. Golden Promise was selected for its desirable agronomic traits such as short stature and earliness, and became a popular malting variety. It was later discovered that Golden Promise also has a more effective Na^+ ^exclusion than Maythorpe in a salt tolerance screening experiment conducted at the Scottish Crop Research Institute [5]. Golden Promise accumulates lower Na^+ ^in shoot tissue compared to Maythorpe under high salt conditions. This ion exclusion was later characterized to be Na^+ ^specific. Golden Promise produced higher number of seeds per plant and fewer sterile seeds than Maythorpe when exposed to salt stress [6].

Besides ion exclusion, other phenotypes which distinguish the mutant were a short stiff straw, semi dwarf stature, a compact inflorescence, short awns and early flowering. This phenotype complex has been attributed to a single recessive mutation called *GPert *that mapped to chromosome 7(5H) of barley near the centromere [7]. *GPert *was reported to be the single known locus at which Golden Promise differed from Maythorpe [8] based on lack of polymorphism from roughly 300 randomly amplified polymorphic DNAs (RAPDs). This locus was subsequently shown to be allelic to the *Ari-e *locus also located on 5H [9]. The *ari-e *mutants in different genetic backgrounds were also semi-dwarf in stature and accumulated less Na^+ ^than their respective parent genotypes [10].

The Golden Promise-Maythorpe genotypic combination provided an attractive model for understanding salt tolerance in barley at the molecular level. Here the progenitor and the presumed single gene mutant differ in salt tolerance as manifested by Na^+ ^ion exclusion, coupled with improved yield of the mutant under saline conditions. Large-scale transcriptome analysis has been employed previously to gain insight into complex phenomena such as salt stress response [11-13]. The availability of a barley array representing ~22,000 transcripts [14] afforded an opportunity to start understanding the differences in transcriptional responses between Golden Promise and Maythorpe and to attempt to identify the genes involved in regulation of salt uptake. Microarray technology has been used in the past to map mutant genes such as *early flowering 3 *(*ELF3*) and *asymmetric leaves 1 *(*AS1*) in *Arabidopsis *using the whole genome arrays [15]. The Affymetrix Barley1 GeneChip is believed to probe roughly half of the genome. Therefore if salt tolerance of Golden Promise has a detectable transcriptional basis, there is a probability of 0.5 that the salt tolerance gene can be observed using the Barley1 GeneChip.

Several approaches using molecular markers such as RAPDs, SSRs and AFLP have been used to describe the isogenic relationship between Golden Promise and Maythorpe [16]. However, recent advances in detection of polymorphism utilize sequence-based approaches that have a far greater marker density. One such method is the use of arrays to detect Single Feature Polymorphisms (SFPs). The SFP approach can derive genotypes as a by-product of expression analysis, detecting genetic polymorphism (difference in hybridization intensities) within the transcribed sequences queried by the array. Such an approach has been used in several organisms including barley, *Arabidopsis *and rice [17-20]. Another recent advance has been the development of a barley SNP-based genetic map using the Illumina Golden Gate Assay [21-23]. This high throughput approach combines high-density BeadArray technology with allele-specific extension covering the polymorphic loci [21]. This technology can be used to scan in parallel roughly 1500 known SNP loci across multiple genotypes. In this study we couple high-density genotyping with differential expression analysis to investigate the basis of salt tolerance of Golden Promise.

## Results

### SFP Analysis for Variation between Golden Promise and Maythorpe

**Figure 1:**
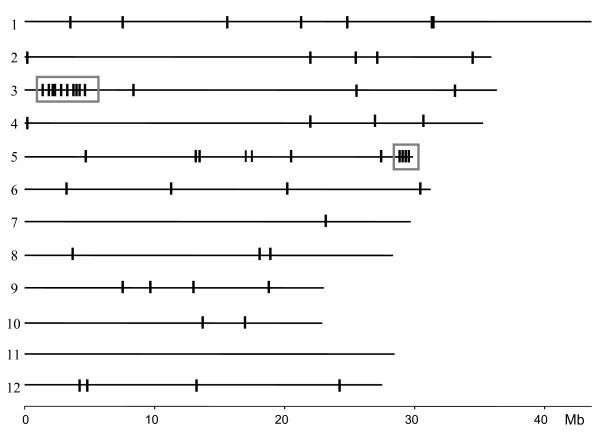
**Rice orthologs of barley genes with single feature polymorphisms (SFPs)**. Rice genome display with rice orthologs (vertical bars) of barley genes which were polymorphic between Maythorpe and Golden Promise. These polymorphic loci were identified using the SFP analysis from the shoot dataset. Two rice gene clusters (boxed) on Chromosome 3 and 5 were identified from the SFP analysis.

The level of polymorphism between Golden Promise and Maythorpe was investigated using the Robustified Projection Pursuit method of SFP analysis which has a validation rate above 80% [18]. Based on a *P*-value cut-off of 0.005, 64 and 46 SFP probe sets were detected from the shoot and root datasets, respectively. The rice orthologs of the barley shoot SFP probe sets were mapped using BLAST and are displayed in Figure 1. Several of the rice orthologs are concentrated within two distinct segments of the rice genome. One is an eleven gene cluster (3.2 Mb) on short arm of chromosome 3 of rice, which is syntenic to barley chromosome 4H. Likewise, a four gene cluster (490 Kb long) was found on the rice chromosome 5 (Figure 1), which is syntenic with barley chromosome 1H. Details of shoot SFPs whose rice orthologs resolved into these two clusters are shown in Table 1. The complete list of SFP probe sets along with the outlying score and position of the SFP probes, and the probe set annotations from HarvEST:Barley are available as supplemental data (Additional files 1, 2, 3 and 4). SFP analysis of Golden Promise versus Maythorpe revealed a low level of polymorphism overall when compared with the other barley genotype comparisons reported in [18]. This is consistent with Golden Promise and Maythorpe being very closely related genetically. However, the SFP analysis established that Maythorpe and Golden Promise differ by more than a single mutation.

### SNP polymorphisms between Golden Promise and Maythorpe

**Figure 2:**
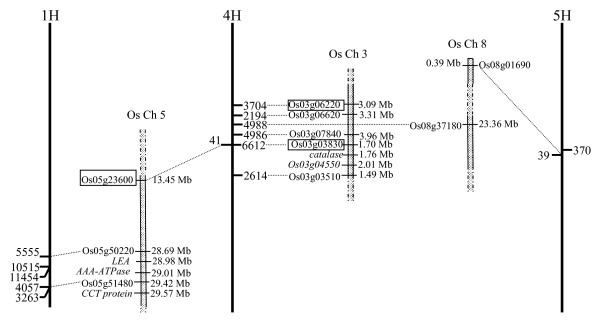
**Polymorphic loci on barley genetic map**. Polymorphic loci between Maythorpe and Golden Promise on barley genetic map and position of corresponding rice orthologs on the genome. The figure shows the polymorphic loci (14 unigenes) between the two genotypes detected by SNP analysis (solid line). The rice orthologs of these barley unigenes derived by BLAST hit to rice database are placed on the left of individual barley chromosomes (1H, 4H, 5H). The rice orthologs of barley genes identified by expression analysis (in italics) and from SFP analysis (boxed) are also shown. This illustrates the regions of haplotype polymorphism between Golden Promise and Maythorpe on barley chromosomes 1H, 4H and 5H.

To more fully gauge the level of polymorphism between Golden Promise and Maythorpe the Illumina OPA genotyping assay was employed. This assay was set-up using 1524 barley SNPs as described in [22]. Each SNP included in the assay has a corresponding "unigene" in HarvEST:Barley derived from assembly 32 [14]. Of the 1524 loci, 1153 were used to generate a barley genetic map. Among these mapped SNPs 14 loci were identified as polymorphic between Golden Promise and Maythorpe. These loci, their corresponding unigenes, and functional annotations found using unigene sequences are listed in Table 2. Two of these loci map to 5H, separted by 3 cM. These two loci on 5H are about 50 cM from the *Ari-e *locus. Seven cluster to a 23.7 cM region of 4H spanning the centromere. The other five loci are within a 6.4 cM cluster on the distal end of the long arm of 1H. The clusters of SNPs on 1H and 4H correspond to the regions identified by SFP analysis, even including some of the same barley genes identified as SFP probe sets. All fourteen SNP loci and their corresponding rice orthologs are displayed in Figure 2.

### Genotype Comparisons at Transcriptome Level

To identify differentially expressed genes between Golden Promise and Maythorpe the crown and growing point tissue and the root tips were sampled from both genotypes growing under control and salinity stressed conditions for transcriptome analysis. The crown and growing point tissue consists of meristematic cells and green growing point obtained by removing the sheath tissue. The term "shoot tissue" is used in this manuscript to describe the crown and growing point tissue. The statistical analysis for differential expression was performed with Significance Analysis of Microarray (SAM) software [24] using three independent biological replicates. Genes that were differentially regulated between the genotypes 25 days after germination are listed in Table 3. Nine probe sets were differentially expressed in shoot tissue at a *q*-value cut-off of 10% (Additional files 5). At the same threshold no differentially expressed probe set in the roots were found. However, fifteen differentially expressed probe sets were identified in roots at a 25% *q*-value threshold (Additional files 6). Three probe sets were shared between the shoot and root comparisons. These include a late embryogenesis protein (*Lea*) and a protein with a CCT (*Co*, *Co-like*, *Toc1*) motif up-regulated in Maythorpe, and Contig6845_at expressing at a higher level in Golden Promise. Probe set Contig6845_at has no sequence match to a known gene. The consensus sequence for this probe set is derived from cDNAs from four different barley genotypes including Golden Promise, providing confidence that it is indeed a barley gene.

Since the phenotypic differences between Maythorpe and Golden Promise were pleiotropic, it was important to ascertain how early in seedling development the difference between the two cultivars at expression level becomes apparent. Therefore the shoot tissue of 10-day old seedlings growing under unstressed conditions from Golden Promise and Maythorpe were sampled for an unreplicated expression analysis experiment. At a two-fold cut-off level, 31 probe sets were differentially expressed. At a more relaxed cut-off of 1.6-fold, 81 probe sets were differentially expressed between the two genotypes. It was observed that seven of the nine probe sets identified from shoot analysis in a fully replicated experiment (Table 3) were also in this list of 81 probe sets. These probe sets are denoted with Y for young shoot analysis in Table 3.

The genotypic array analysis was extended by using the barley microarray reference dataset [25]. This dataset was generated from two barley genotypes, Morex and Golden Promise, using a diverse series of tissues and is available from Plant Expression Database (PLEXdb). The rationale for looking at the Golden Promise-Morex data was that any expression difference observed between Golden Promise and Maythorpe due to gamma-ray treatment is also likely to emerge from a comparison of Golden Promise with other genotypes including Morex. A genotypic comparison of Golden Promise with Morex yielded 955 probe sets as differentially expressed when comparing the same tissue types as in this study. Stringent statistical analysis using SAM was also employed on this triplicate dataset for differential expression. On comparing the Golden Promise-Morex probe set list to the Golden Promise-Maythorpe list in Table 3, six of the nine probe sets identified in the shoot Maythorpe-Golden Promise comparison were found in common with Morex-Golden Promise comparison (denoted with M). Six of the 15 probe sets identified from the Maythorpe-Golden Promise root tissue comparison were also identified in the Golden Promise-Morex comparison. These probe sets which were derived from intersection of two genotypic comparisons constitute a more robust list of genes for differential expression in Golden Promise than would a list derived from only the Golden Promise-Maythorpe comparison.

**Figure 3:**
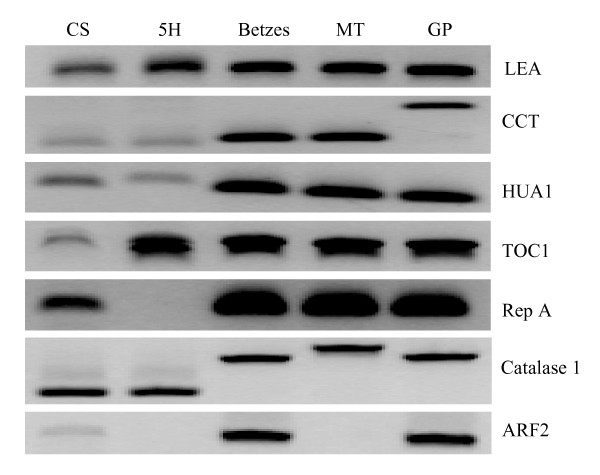
**Differentially expressed genes and 5H wheat-barley addition line**. Some of the differentially expressed genes were checked for map position on chromosome 5H of barley (*Ari-e *locus is on 5H). Wheat background genotype used is Chinese Spring (CS), the barley genotype Betzes, and the addition line is 5H. None of the genes identified by array analysis (*LEA*, CCT motif protein, *HUA1*, *Replication protein A*, *catalase1*, and *ARF2*) mapped to 5H. A gene which maps to 5H (e.g. Timing of CAB expression 1, *TOC1*) is expected to have a stronger band in 5H relative to CS. Three genes *CCT, catalase 1 *and *ARF2 *have different alleles in Maythorpe (MT) and Golden Promise (GP).

**Figure 4:**
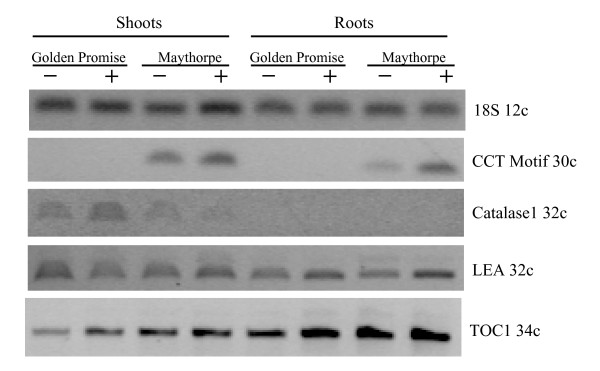
**RT PCR validation of some key genes**. Expression level of some genes discussed in the current study were validated by RT-PCR for shoot and root tissues. The (-) indicates control unstressed samples and (+) indicates salinity stressed samples.

Since the *GPert *mutation in Golden Promise was previously mapped to barley chromosome 5H as an allele of the *ari-e *locus [7], we initially used wheat-barley addition line for 5H [26] to determine if any of the regulated genes identified from array analysis map to 5H. Six genes encoding LEA protein, CCT motif family protein, *Hua1*, replication protein A (*RepA*), *Catalase1 *and auxin response factor (*Arf2*) from Table 3 were selected. Selection of these genes was based on their identification from two or more expression-based genotypic comparisons involving 10-day and 25-day Golden Promise-Maythorpe comparisons and the Golden Promise-Morex comparisons. The 5H addition line consists of disomic 5H from Betzes barley in the Chinese Spring hexaploid wheat background (Figure 3). None of these genes mapped on 5H, consistent with SFP and SNP analysis. Furthermore, size polymorphisms in the amplicons for CCT motif protein, and catalase1 and a missing band for Maythorpe (presence/absence polymorphism) in the case of *Arf2 *were detected. These amplicon size polymorphisms indicated that these three genes were not only differentially expressed but were in different allelic forms in the two genotypes. The difference in expression for CCT motif protein and catalase1 was validated with semi-quantitative RT-PCR (Figure 4).

### Differential Expression in Response to Salinity Stress

**Figure 5:**
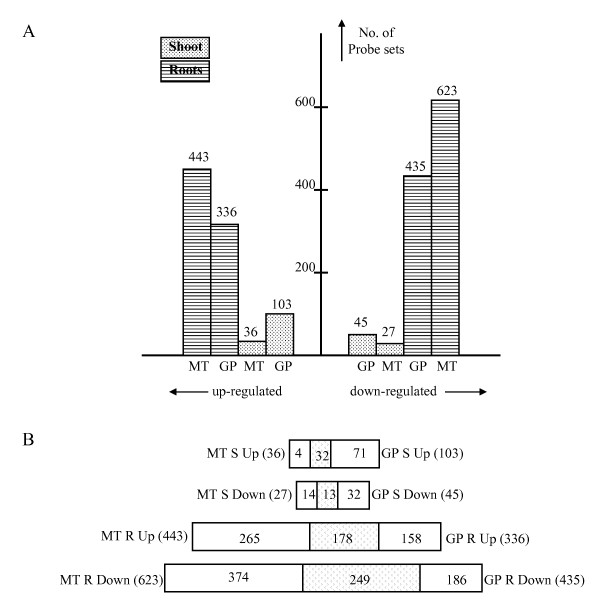
**Salinity stress responsive genes in Maythorpe and Golden Promise**. A. Number of probe sets up- and down-regulated in root (horizontal lines) and shoot tissue (dots) of the two genotypes. B. Venn diagram stating the overlap between the up- and down-regulated probe sets in Maythorpe (MT) and Golden Promise (GP). The overlap between the two genotypes is shaded with dots. The total number of probe sets responding in each the genotypes is indicated in brackets ().

How different are the salinity stress responses of two genotypes which are genetically very similar but differ in salt-tolerance? How do the transcriptional responses of roots compare to those of shoot under salinity stress for a given genotype? To address these questions, the shoot and root samples from control and stressed conditions at 25-day time point from Golden Promise and Maythorpe were compared. Differential expression analysis was performed using SAM and the false discovery rate (FDR) was controlled to be below 15%. Lists of differentially expressed genes in response to salinity stress observed in Golden Promise and Maythorpe are provided as additional files (7 to 14). The number of probe sets responding significantly to salinity treatment for each of the genotype and tissue combinations is shown in Figure 5A. The results show that a higher number of genes are salt stress regulated in the roots compared to the shoot tissue in both genotypes. Additionally, roots in both genotypes responded by down-regulation of more genes than by up-regulation. The salinity stress response of Golden Promise is significantly different compared to Maythorpe (Figure 5B). This difference is more apparent in the root comparisons.

These results indicate that the response of roots to salinity stress at the transcriptional level is very different from the shoots in both genotypes. Only 16 and 9 probe sets were found to be commonly induced between roots and shoots in Golden Promise and Maythorpe, respectively. Of these, 4 probe sets were induced in the roots and shoot tissue of both genotypes. The probe sets represent delta-l-pyrroline-5-carboxylate synthetase, a lipid transfer protein, phosphoethanolamine cytidyltransferase and barley dehydrin 7. Three of these genes are associated with abiotic stress response in plants.

## Discussion and Conclusion

### Genetic Polymorphism between Golden Promise and Maythorpe

The results presented clearly show that Golden Promise and Maythorpe are polymorphic at multiple loci. Fourteen polymorphic SNP loci resolve into three clusters on 1H, 4H and 5H (Fig. 2), two of which (1H and 4H) were also found by SFP analysis (Fig. 1) and by the position of loci with amplicon size polymorphisms (Figs. 2 and 3). These two genotypes were previously reported to be isogenic differing at a single locus, *GPert *on 5H [6,16,27]. Certainly this is not the only difference between the accessions of Maythorpe and Golden Promise that we analyzed. A different accession of Maythorpe with a slightly different genetic constitution, reflecting residual polymorphism within the cultivar, probably was used as the parent of Golden Promise. The polymorphism on 5H is at about 50 cM distance from the *Ari-e *locus, so the presence of this 5H haplotype block in the accession of Maythorpe that was examined is still consistent with a mutational origination of Golden Promise from Maythorpe.

None of the probe sets representing the 14 SNP loci were found to be differentially expressed in the genotypic comparisons in both tissue types. Additionally, the 14 probe sets were found to not respond to salt stress in the two genotypes. Some of the rice orthologs of barley probe sets identified as differentially expressed in genotypic comparisons localized in the vicinity of polymorphic haploblocks (Figure 2). Seven polymorphic SNP loci between Golden Promise and Maythorpe clustered around the 4H centromere and 5 of these corresponded to an orthologous rice region spanning 2.5 Mb of chromosome 3. Two loci (including *Catalase1*) were found to be differentially expressed from the GeneChip analysis. The segment on the long arm of 1H and its corresponding rice chromosome 5 segment (<1 Mb) also emerged as different between Golden Promise and Maythorpe. This segment includes the rice orthologs of the CCT motif protein and the *Lea *protein besides an AAA-type ATPase. Both CCT and *Lea *protein encoding genes were the only two genes consistently identified from differential expression analysis involving Golden Promise, Maythorpe and Morex in all tissue types and stages. The differential expression of AAA-type ATPase is important in context of a recent report which characterized an ice plant AAA-Type ATPase gene, SKD1 and suggested a role in compartmentalization of excess Na^+ ^[28].

### Golden Promise Phenotype and Regulated Genes

The genes identified by the genotypic analyses performed in this study (Table 3) do not have an obvious functional association with the favorable Na^+ ^homeostasis maintained by Golden Promise. No known Na^+ ^transporters conferring this trait were identified. Two possible explanations can be proposed for this: 1) some of the genes/loci identified have no annotation, or have sequence match to an uncharacterized expressed protein; these uncharacterized genes could be regulating ion homeostasis 2) the Barley1 GeneChip does not probe the transcript which can be directly associated with favorable ion homeostasis in Golden Promise. All the expression and polymorphism based approaches used in this study are directly or indirectly derived from the EST sequence assembly probed by the Barley1 GeneChip. Our analysis did not query every gene in barley genome.

Low Na^+ ^accumulation has been associated with early flowering genes in *Triticeae *[29,30]. It is noteworthy that Golden Promise was reported to flower earlier than Maythorpe [16]. Early flowering in Golden Promise was also observed under our experimental conditions. Our expression analysis identified a gene with CCT motif as differentially expressed between Golden Promise and Maythorpe. The CCT motif is present in several genes known to regulate flowering time [31]. It raises the possibility of the CCT motif belonging to a repressor of flowering, which is down-regulated in Golden Promise. Two other genes which emerged from the expression analysis are known regulators of inflorescence/flower architecture. First is *Hua1 *which is up-regulated in Golden Promise in young, 10-day as well as 25-day old plants in the shoot tissue. *Hua1 *is an RNA binding protein which is involved in flower development in *Arabidopsis *[32]. The second gene encodes an auxin response factor 2 (*Arf2*). A mutation in *Arf2 *in *Arabidopsis *is known to result in pleiotropic effects on the phenotype [33-35]. The *arf2 *mutants are reported to have increased seed size and larger aerial organs, delayed flowering and leaf senescence among other phenotypes.

Intriguingly, some of the phenotypes distinguishing Golden Promise from Maythorpe include small seed size, and decreased plant height, compact inflorescence and early flowering [16]. It is pertinent to point out that none of these three genes map to chromosome 5H of barley where the original mutation (*ari-e*) is mapped. Therefore, if these genes control the observed phenotypes such as flowering time difference and seed size variation, then the differences cannot be attributed to *Ari-e *locus on 5H.

### Differential Response to Salinity Stress

Considering the genetic difference between Maythorpe and Golden Promise in regulation of shoot Na^+ ^homeostasis under salt stress, we found the differential expression of two cation transport related genes to be particularly interesting. A Na^+^/Ca^2+ ^exchanger protein (Contig4515_at) was down-regulated in Maythorpe roots in response to salinity stress (*q*-value, 9.4%). Supplemental Ca^2+ ^is known to reduce Na^+ ^influx in plant [36,3]. The down-regulation of the Na^+^/Ca^2+ ^exchanger in Maythorpe but not in Golden Promise may explain the low Na^+ ^accumulation trait of Golden Promise as well as the supplemental Ca^2+^-linked Na^+ ^efflux reported by several researchers. Another gene with a similar expression profile encodes a vacuolar cation/proton exchanger (Contig4212_s_at). This gene has a sequence match in *Arabidopsis *database to a calcium proton antiporter, *Cax3*. It is a Ca^2+ ^exchanger predominantly active in root tonoplasts and is required for growth and nutrient acquisition [37].

Jasmonic acid (JA) related genes were differently regulated in the two genotypes. Several of the JA biosynthetic pathway genes were down-regulated in response to salinity in Maythorpe. These included 12-oxophytodienoate reductase 2 (*Opr2*), allene oxide synthase (*Aos*), and lipoxygenases (*Lox2 and Lox3*). In contrast, biosynthesis gene allene oxide cyclase (*Aoc*) and two jasmonic acid-induced proteins (JIPs) were up-regulated in Golden Promise but not in Maythorpe in response to stress (Table 4). The allene oxide synthase (*Aos*) gene represented by Contig3097_at (Unigene 2094) on the array was found to have an SFP (P < 0.05) between Golden Promise and Maythorpe. Interestingly, this gene maps to the haploblock on 4H at 61.7 cM. Differential regulation of jasmonic acid related genes between the two genotypes can potentially be due polymorphism at the *Aos *gene which lies on the 4H haploblock. It has been reported previously that JA-pretreatment improves salinity stress adaptation in barley [38]. Recent experiments from our laboratories have demonstrated that JA-pretreatment of barley plants before salinity stress induced JA biosynthesis genes and improved salt-tolerance by maintaining lower shoot Na^+ ^relative to stressed plants with no pretreatment [39]. It will be interesting to investigate if the differential regulation of JA-related genes in low Na^+ ^accumulating Golden Promise and association of JA-pretreatment with Na^+ ^exclusion is purely coincidental. If not, JA appears to be an important component of heritable salt tolerance in Golden Promise and barley in general.

## Methods

### Plant Materials and Experimental Conditions

Barley seeds [*Hordeum vulgare *L. cv. Golden Promise (spring barley)] were initially provided by Peggy Lemaux (University of California, Berkeley). Maythorpe seeds were obtained from the National Small Grains Collection, Idaho. Seed stocks were multiplied in the field at the University of California, Riverside. Seeds were washed several times with deionized water and germinated on moistened filter paper in glass crystallization dishes for two days in darkness. The plants were grown in a greenhouse at U.S. Salinity Laboratory, USDA-ARS, at Riverside, California in September and October, 2004. Germinated seeds were transferred onto Speedling Trays floated on aerated half-strength Hoagland's solution, with double iron (50 gL^-1^) in 700 L metal containers. The pH was maintained within the range of 5 to 6.5 using concentrated sulfuric acid. Electrical conductivity, pH and solution temperature were monitored daily.

On day 16 after germination (3–4 leaf stage), a salinity stress of was imposed over a period of five days in five equal steps to reach a final concentration of 17 dS m^-1 ^(~150 mM NaCl). CaCl_2 _was added with NaCl to maintain a 10:1 molar ratio of Na^+^: Ca^2+^. The system was allowed to stabilize for five days. On day 25 (5–6 leaf stage) "shoot" (crown and growing point) tissue and root (2 cm of the root tips) tissue from 15 plants was harvested and snap frozen for RNA extraction. Therefore tissue from 15 plants from each genotype per tank constituted a single replicate of a treatment. Three biological replicates of the experiment were sampled.

### Phenotypic Measurements

Before proceeding with the expression studies, the reported Golden Promise and Maythorpe salt stress response phenotype for difference in Na^+ ^accumulation in the shoot tissue under our growing conditions was tested. Whole shoot tissue from six plants was pooled to form each replicate. Seven replicates were collected from each of the four treatments. Plants were washed with deionized water, dried in a forced air oven (70°C) then ground into fine powder. Shoot Na^+ ^concentrations were determined on nitric-perchloric acid digests by inductively coupled plasma optical emission spectrometry (ICP, Perkin-Elmer Co., Norwalk, CT, USA). The results from the shoot ion analysis confirming the ion exclusion phenotype of Golden Promise are listed in Table 4.

### Target Preparation and Processing for GeneChip Analysis

RNA samples were processed as recommended by Affymetrix, Inc. (Affymetrix GeneChip Expression Analysis Technical Manual, Affymetrix, Inc., Santa Clara, CA) at the DNA and Protein Microarray Facility at University of California, Irvine. Total RNA was initially isolated from frozen shoot tissue using TRIzol Reagent. The RNA was purified by passing through an RNAeasy spin column (Qiagen, Chatsworth, CA) and on-column DNaseI treatment. Eluted total RNAs were quantified with a portion of the recovered total RNA and adjusted to a final concentration of 1 μg/μl. Labeling and hybridization were performed at the DNA and Protein Microarray Facility at University of California, Irvine. All starting total RNA samples were quality assessed prior to beginning target preparation/processing steps by running out a small amount of each sample (typically 25–250 ng/well) onto a RNA Lab-On-A-Chip (Caliper Technologies Corp., Mountain View, CA) that was evaluated on an Agilent Bioanalyzer 2100 (Agilent Technologies, Palo Alto, CA). Single-stranded, then double-stranded cDNA was synthesized from the poly(A)+ mRNA present in the isolated total RNA (10 μg total RNA starting material each sample reaction) using the SuperScript Double-Stranded cDNA Synthesis Kit (Invitrogen Corp., Carlsbad, CA) and poly (T)-nucleotide primers that contained a sequence recognized by T7 RNA polymerase. A portion of the resulting ds cDNA was used as a template to generate biotin-tagged cRNA from an *in vitro *transcription reaction (IVT), using the BioArray High-Yield RNA Transcript Labeling Kit (T7) (Enzo Diagnostics, Inc., Farmingdale, NY). Fifteen μg of the resulting biotin-tagged cRNA was fragmented to strands of 35–200 bases in length following prescribed protocols (Affymetrix GeneChip Expression Analysis Technical Manual). Subsequently, 10 μg of this fragmented target cRNA was hybridized at 45°C with rotation for 16 hours (Affymetrix GeneChip Hybridization Oven 320) to probe sets present on an Affymetrix Barley1 array (Close et al. 2004). The GeneChip arrays were washed and then stained (SAPE, streptavidin-phycoerythrin) on an Affymetrix Fluidics Station 400, followed by scanning on a Hewlett-Packard GeneArray scanner.

### Data Analysis

The scanned GeneChip images were examined for any visible defects. Satisfactory image files were analyzed to generate raw data files saved as .CEL files using default settings of GeneChip Operating Software (GCOS 1.2, Affymetrix). The .CEL files from replicated data sets were imported into RMA [40] for background adjustment and quantile normalization. The log-transformed RMA values for all probe sets were imported into Significance Analysis of Microarrays (SAM) software [24] using the two-class unpaired data format. For genotypic comparisons (for instance, a control GP vs. MT) we initially set a permutation-based false discovery rate (FDR) cut-off (expressed as *q*-value) at 10%. At this initial threshold, we did not find any probe set to be differentially expressed in the root comparison (GP control roots vs. MT control roots). Therefore, the threshold was relaxed to 25% (Additional files 5 and 6). For differential expression analysis in response to salinity stress, a threshold of 15% was used.

For analysis of the single replicate dataset generated for basal gene expression levels in 10-day old seedlings of both genotypes, DChip was used [41]. DChip was set to import GCOS signals. Normalization of the datasets was performed using an invariant-set approach. To calculate the expression index of probe sets we used the PM model. After expression values were computed, genes with extremely low values were assigned a value equivalent to the average value of the lowest 10^th ^percentile of all the genes that are called absent. This step prevents the overestimation of fold changes for very weakly expressed genes. The expression values were log_2 _transformed after calculating the expression index. Differentially expressed probe sets were identified using a fold change cut-off criteria for up-regulation or down-regulation.

Single Feature Polymorphisms (SFPs) analysis was performed as described in [18]. Since this method uses RNA as a surrogate for genomic DNA for hybridization to the arrays, we used the data obtained from RNA hybridization of roots and shoot tissue for the analysis. Root and shoot data were analyzed separately. The probe sets identified from the analysis at a P-value cutoff of 0.005 are listed in (Additional files 1 and 2).

### Probe Set Annotations and Gene Ontology Analysis

The probe sets were annotated using HarvEST:Barley (version 1.47) assembly 21 [42]. The output from HarvEST included the best BLAST hit from TIGR translated rice gene models (version 4) and TAIR translated *Arabidopsis *gene models. Besides a description of the best hit, output also includes the genome location (chromosome and base pair position) of the best BLAST hit gene models in rice and *Arabidopsis*.

### Expression validation by semi-quantitative RT-PCR

Expression profiles of several key transcripts obtained from chip hybridizations were further validated by semi-quantitative RT-PCR using first strand cDNA synthesis from RNA samples. A cDNA first strand was synthesized using Taq-Man Reverse Transcription Reagents (Applied Biosystems, Forster City, CA; Ref: N808-0234) following the manufacturer's instructions. Two micrograms of total RNA was converted into cDNA. Each cDNA was diluted 40 fold and 5 μL of cDNA was used for PCR. A 18s ribosomal RNA (forward: atgataactcgacggatcgc; reverse: cttggatgtggtagccgttt; cycles) was used as control for RT-PCR experiments.

### Data Availability

All expression data will be made available through the Gene Expression Omnibus (GEO) under platform GPL1340, Series GSE6325. The list of significantly responsive probe sets along with annotations is available as Additional Files. The enhanced annotation for all Barley1 probe sets is available through HarvEST:Barley [42].

## Authors' contributions

HW contributed in the design of the experiment, cultured the plants, analyzed the array data, and drafted the manuscript. CW designed the experiment, cultured the plants, performed ion analysis, and provided significant input to the manuscript. PC performed RT-PCR and wheat-barley addition line analysis. AMI is the co-principal investigator on the project and had significant input in the design of the experiment. JX and XC performed the SFP analysis. TJC is the principal investigator of the grants that funded the project and had input in the design of experiment, conducted the SNP polymorphism analysis and helped write the manuscript.

## Supplementary Material

Additional file 1 Shoot SFP GP vs MT scores Click here for file

Additional file 2 Root SFP GP vs MT scores Click here for file

Additional file 3 Shoot SFP probe sets Click here for file

Additional file 4 Root SFP probe sets Click here for file

Additional file 5 GPcontrol vs MTcontrol shoot Click here for file

Additional file 6 GPcontrol vs MTcontrol root Click here for file

Additional file 7 GPcontrol vs GPsalt shoot up-reg Click here for file

Additional file 8 GPcontrol vs GPsalt shoot down-reg Click here for file

Additional file 9 GPcontrol vs GPsalt root up-reg Click here for file

Additional file 10 GPcontrol vs GPsalt root down-reg Click here for file

Additional file 11 MTcontrol vs MTsalt shoot up-reg Click here for file

Additional file 12 MTcontrol vs MTsalt shoot down-reg Click here for file

Additional file 13 MTcontrol vs MTsalt up-reg root Click here for file

Additional file 14 MTcontrol vs MTsalt root down-reg Click here for file

## Figures and Tables

**Table 1:** List of selected barley SFPs and their rice orthologs which resolve into tight clusters

**SFP Probe Set**	**Outlying Score**	**Unigene**	**Rice Locus**	**Rice Locus 5' in Mb**
Contig8980_at	22.4	6602	Os03g03390	1.423341
Contig14557_at	26.6	11753	Os03g03410	1.435869
Contig15294_at	19.7	12479	Os03g03460	1.475590
Contig8880_at	39.5	6612	Os03g03830	1.706250
EBro08_SQ004_F05_at	47.4	27411	Os03g04550	2.102803
Contig2034_s_at	17.4	1375	Os03g05730	2.835792
Contig5198_s_at	22.6	3704	Os03g06220	3.095097
Contig10796_at	22.2	8498	Os03g07800	3.949327
Contig5720_at	31.6	4133	Os03g07870	3.980770
Contig13638_at	17.1	11204	Os03g07970	4.056735
Contig6015_at	16.8	4388	Os03g08940	4.614255
Contig11667_at	27.1	9022	Os05g50800	29.054191
Contig4593_at	19.3	3245	Os05g50810	29.061250
Contig11883_at	45.0	9212	Os05g50970	29.165814
Contig14126_at	30.5	10804	Os05g51650	29.539790

**Table 2:** List of barley polymorphic SNP unigenes between Golden Promise and Maythorpe

**SNP Unigene**	**Barley Group**	**Rice Locus**	**Putative Function**	**SFP Probe Set**
39	5H	Os08g01690	transposon protein	-
3263	5H	Os04g06770	Piwi domain containing protein	-
370	5H	Os09g30340	Photosystem I reaction center V	-
2614	4H	Os03g03510	CIPK-like protein 1	-
6612	4H	Os03g03830	EF hand family protein	Contig8880_at
3704	4H	Os03g06220	ATP-dependent RNA helicase	Contig5198_s_at
2194	4H	Os03g06620	1,2-dihydroxy-3-keto-5-methylthiopentene dioxygenase	-
4986	4H	Os03g07840	Adenylosuccinate synthetase	-
41	4H	Os05g23600	Ribophorin I family protein	Contig4748_s_at
4988	4H	Os08g37180	Patatin-like phospholipase protein	-
5555	1H	Os05g50220	Peroxisomal membrane anchor protein	-
4057	1H	Os05g51480	DNA damage binding protein 1a	-
10515	1H	Os01g74020	myb-like DNA-binding domain	-
11454	1H	Os01g42960	TPR Domain containing protein	Contig14590_at

**Table 3:** List of probe sets which are differentially expressed between Golden Promise and Maythorpe under unstressed

Probe set ID	Putative Function	Rice Chr.	5' Rice Gene	Expression & Criteria	*q*-value %	fold change
**Root Comparison**						
Contig8052_at	splicing factor PWI domain protein	3	29429795	MT up; R	25	2.0
Contig3842_at	LEA protein	5	28987287	MT up; RSM	25	34.3
Contig6165_at	CCT motif protein	5	29573293	MT up; RSM	25	9.9
Contig15475_at	reticuline oxidase precursor	6	20743699	MT up; R	25	6.1
Contig23396_at	expressed protein	-	-	MT up; RM	25	2.6
Contig6845_at	no hit	-	-	GP up; RS	25	2.9
Contig2510_at	expressed protein	-	-	GP up; R	25	1.8
Contig25937_at	structural maintenance of chr (SMC)	-	-	GP up; R	25	1.7
Contig17352_at	expressed protein	-	-	GP up; R	25	1.7
HVSMEn0005L15f	expressed protein	-	-	GP up; R	25	1.7
Contig18699_at	zinc finger (C3HC4-type RING finger)	1	39965630	GP up; R	25	1.8
Contig7273_s_at	replication protein A2	2	25402499	GP up; R	25	5.0
Contig25428_at	RPA 32 kDa	2	25402499	GP up; RM	25	2.6
Contig24975_at	expressed protein	4	10975293	GP up; R	25	1.7
Contig10006_at	AAA-type ATPase family protein	5	29015582	GP up; R	25	2.2
**Shoot Comparison**						
Contig3842_at	LEA protein	5	28987287	MT up; SRYM	10	14.4
Contig6165_at	CCT motif protein	5	29573293	MT up; SRYM	10	5.7
EBro08_SQ004_F05	expressed protein	3	2102803	MT up; SY	10	2.8
HV_CEb0003E19r2	Auxin response factor 2	1	41022513	MT up; SM	10	2.0
Contig6845_at	no hit	-	-	GP up; SRM	10	10.7
S0000800234C01F1	expressed protein	7	5429853	GP up; SYM	10	3.3
HX01D24w_at	expressed protein	7	5429853	GP up; SYM	10	1.5
Contig1846_s_at	Catalase1	3	1769149	GP up; SY	10	3.4
Contig11254_at	floral homeotic protein (HUA1)	1	24805279	GP up; SY	10	3.4

R: root GP-MT comparison of 25-day old plants; S: shoot GP-MT comparison of 25-day old plants; Y: shoot GP-MT comparison of 10-day old plants; M: multiple

**Table 4:** The main shoot Na^+^concentrations of Maythorpe and Golden Promise after 9 d under salinity stress

	Maythorpe	Golden Promise
Control	68.2 ± 5.1	81.1 ± 5.6
Salt Stress	1211.1 ± 48.8	958.8 ± 64.7

Mean values of 7 replicates ± SE. The Na^+ ^concentration is expressed in mmolKg^-1 ^dry weight.
